# Annual Research Review: Improving school climate to improve child and adolescent mental health and reduce inequalities

**DOI:** 10.1111/jcpp.70061

**Published:** 2025-10-12

**Authors:** Graham Moore

**Affiliations:** ^1^ DECIPHer, School of Social Sciences Cardiff University Cardiff UK; ^2^ Wolfson Centre for Young People's Mental Health Cardiff University Cardiff UK

**Keywords:** School, intervention, mental health

## Abstract

Schools are important settings for intervention to improve mental health. Much school mental health research has focused on schools as an avenue to reach large numbers of young people with new interventions, added on top of what schools currently do. However, research is increasingly focused on changing the school system itself to improve mental health, with a growing emphasis on improving school climate. This article begins by exploring wider debates on the benefits and harms of school‐based interventions, before focusing on school climate as a target for intervention. It reviews evidence from intervention studies and systematic reviews to understand effectiveness, how interventions reduce or amplify inequalities, and real‐world impacts. School climate research has grown rapidly since the turn of the century. It remains difficult to define. Definitions vary in whether they include focus on physical environments and educational instruction. However, they converge on focus on positive relationships among a school community and safety. Several large trials of interventions to improve mental health, by improving school climate, have been conducted in a range of international contexts. While many have not been effective, recent trials provide evidence that interventions *can* improve school climate and mental health, as well as a range of risk behaviours. Few studies examine effects on inequalities in mental health, with tentative evidence that school climate interventions have been more effective for some groups than others (e.g., bigger effects for boys than for girls). Evidence on scalability and sustainability indicates that typically small effects from trials may not fully translate into real‐world change. There is growing evidence that improving school climate interventions can improve child and adolescent mental health. More research is needed on how such interventions can contribute to reducing inequalities. Further work is needed to understand how effects translate into real‐world public health impact.

## Introduction

Childhood and adolescence are vital periods in life‐course mental health trajectories, with most mental health difficulties emerging before age 25 (Solmi et al., 2022). Emotional difficulties, including symptoms associated with depression and anxiety, have risen substantially in the past decade (Anthony et al., 2024; Slee, Nazareth, Freemantle, & Horsfall, 2021). This has likely been driven by factors including the global financial crisis (Slee et al., 2021), and accelerated by the COVID‐19 pandemic (Moore et al., 2022). Alongside high levels of population mental health difficulties, there is substantial inequality in child and adolescent mental health along diverse individual and intersecting characteristics, such as gender, ethnicity, and socio‐economic status (Harwood, Newman, Moore, Lee, & Anstey, 2021).

Schools are commonly seen as important channels for preventing mental health problems, promoting positive mental health, and reducing inequalities. UK nations have developed a range of guidance for schools on adopting a ‘whole‐school approach’ to mental health and well‐being (Public Health England, 2015; Welsh Government, 2021), while the World Health Organisation advocates for a range of school‐based interventions to improve mental health (Ross et al., 2021). This is in part because, in societies with universal education provision, schools provide access to almost entire populations. This makes them ‘ideal’ settings for identifying those who would benefit from targeted interventions, and for universal interventions to improve mental health across populations (Werner‐Seidler et al., 2021).

An important caveat in viewing schools as an optimal channel for intervention is that school non‐attendance (McDonald, Lester, & Michelson, 2023) and education outside of school (Alikhani, 2024; Banks, Forlin, & Chambers, 2023) have increased substantially. Perhaps, interventions to improve school experiences are one way to make school easier for young people to engage with (Boaler & Bond, 2023). In the meantime, intervening solely within schools may miss a growing sector of the population, potentially widening health inequalities (Forlin & Chambers, 2023).

Beyond seeing schools as convenient ways of reaching the population, movements to intervene via schools also increasingly recognise that school climate itself (i.e. the overall feel and atmosphere of a school shaped by how staff and students interact and how safe people feel) has the potential to improve, and in some cases harm mental health. Consistent with Ottawa Charter principles for Health Promotion (World Health Organization, 1995), school‐based interventions are increasingly designed around changing a major setting in which young people ‘live, work, love, and play’.

While the primary focus of this article will be on school climate, it will first explore broader debates on the role of schools and school‐based intervention in improving (or harming) young people's mental health. It will argue that schools have a role to play within societal efforts to improve young people's mental health, while recognising the importance of being realistic about what schools can do, and considering unintended consequences. Subsequently, this article will introduce the concept of ‘school climate’, including a range of related terms and definitions. It will discuss evidence from intervention studies and systematic reviews on the effectiveness of interventions in improving school climate and mental health. It is beyond the scope of this article to provide a new systematic review. It will provide a detailed overview of several case examples and recent systematic reviews. It will then discuss the need to understand how school climate interventions may reduce or amplify inequalities in mental health. Finally, this article will focus on real‐world impacts.

In summary, the aims of this article are:

- To introduce key current debates on the potential benefits and harms of school‐based interventions to improve mental health.
- To define school climate as a target for school‐based intervention and consider evolving evidence for the effectiveness of interventions in improving school climate and mental health.
- To consider how school climate interventions may reduce or amplify mental health inequalities, and their likely real‐world impacts.

## How much benefit, or harm, can we expect school‐based mental health interventions to do?

### ‘School effects’ on mental health

In much early school effects literature, a common means of estimating these was to examine intra‐cluster correlations (Sellström & Bremberg, 2006; West, Sweeting, & Leyland, 2004). These are a statistical indicator of the extent to which two individuals from the same cluster (i.e., school) are more similar than two drawn at random from the population, after adjusting for compositional effects. Compositional effects refer to differences between schools arising from who attends the respective schools (Herke et al., 2022). Once accounted for, remaining clustering is presumed to reflect differences between schools. Of course, ‘fully accounting for’ compositional differences is a hypothetical condition which can never be achieved. Further, issues of reverse causality are often under‐considered in multilevel modelling. Schools with happier pupils, for example, may attract certain types of families and pupils, leading to changes in composition.

Multilevel analyses across a range of international contexts indicate that a small portion of variation in psychosocial health outcomes is explained by differences between schools (Shackleton, Hale, Bonell, & Viner, 2016). Intra‐cluster correlations are lower for mental health outcomes than for socially contagious behaviours, such as smoking, with around 2% of variation in depressive mood occurring at the school level (Shackleton et al., 2016). Our surveys in Wales indicate that intra‐cluster correlations may be slightly higher in primary schools, though still only 3%–4% of variation occurs at the school level (Donaldson, Morgan, Ouerghi, Lewis, & Moore, 2025; Moore et al., 2024).

However, understanding the effects of schools on mental health requires considerations beyond between school‐level variability. Many characteristics of schools which have implications for children's mental health are reflected across whole education systems, rather than primarily varying between schools within them. Chris Bonell (2025) argues that many contemporary harms of schools in England derive from national drivers towards narrow pedagogy, unflexible disciplinary systems, and didactic undifferentiated forms of teaching. In Wales, time trends in adolescent emotional difficulties mirror rising perceptions of school pressure across the population (Armitage et al., 2025). In Sweden, declines and widening inequalities in school belonging coincided with whole‐system performance‐based reforms (Högberg, Petersen, Strandh, & Johansson, 2021). Hence, across a range of national education systems, schools appear to be becoming an increasing source of emotional difficulty and a weaker source of social connection for many young people.

In addition, comparisons *between* schools offer limited insight into variations in experience *within* schools. Individual experiences of the same school are often highly patterned by a range of axes of inequality, such as gender, ethnicity, and socio‐economic status (Harwood et al., 2021). Morrison Gutman and Feinstein (2008) argue that a focus on ensuring child‐school ‘fit’ across the whole of a school's diverse population may in many cases be more important than choosing a particular school.

Hence, a holistic understanding of school effects requires us to look beyond variations between schools. It requires us to understand how whole education systems act to improve or harm mental health. Addressing variations in individual and subgroup experiences of school is also vital to mitigating risks of schools reproducing inequalities within the societies in which they operate, through providing environments that offer a better ‘fit’ for some groups of pupils than others (Bernstein, 2003; Bourdieu, 2018).

### Targeted and universal interventions in schools

School‐based interventions to improve mental health and reduce inequalities may be targeted toward at‐risk groups or fully universal. There is some evidence that targeted interventions for depression and anxiety produce larger effects per individual for small subsets of the population (Werner‐Seidler et al., 2021). Targeted interventions explicitly aim to reduce inequalities, focusing resources on those at greatest risk.

However, while targeted interventions are important, they may have unintended consequences, including introducing or reinforcing stigma (Evans, Scourfield, & Murphy, 2015). Targeting individuals who meet a threshold may lead to the exclusion of a ‘missing middle’ who are struggling but not to the extent that meets thresholds for receipt of intervention (Dunn et al., 2024). School‐based interventions may be targeted at the group level, including providing intervention only to schools with disadvantaged intakes. However, this risks missing young people from less affluent families attending schools where most others are more affluent than they are; a configuration associated with particularly poor well‐being (Moore et al., 2017). As mental health difficulties rise throughout populations, targeted interventions are also unlikely to reach the growing number of young people who may benefit from them.

Many mental health difficulties are dimensional rather than binary, and hence intervention in schools may benefit from proportionate universalist approaches (Birrell et al., 2025). While commonly defined in relation to varying levels of socio‐economic disadvantage, these are approaches in which actions are universal, but with intensity and scale varied in proportion to need (Marmot & Bell, 2012). While examples are available from neighbourhood‐level interventions to reduce child health inequalities (Egan et al., 2016), in schools as in wider public health policy, this approach has proven difficult to operationalise in practice to date (Francis‐Oliviero, Cambon, Wittwer, Marmot, & Alla, 2020).

Schools also represent an important avenue for fully universal approaches which aim to achieve improvements across populations, often through small average effects per participant (Rose, 2001). While many mental health interventions within schools have failed to improve outcomes (Caldwell et al., 2019; Mackenzie & Williams, 2018), as we will discuss, some have found small but worthwhile effects. It is important to be realistic about the effects we expect to see, given that much variation in mental health is driven by factors over which schools have little control. It is also crucial to consider whether population‐level improvements have the potential to reduce or widen inequalities (Moore, Littlecott, et al., 2015). Nevertheless, small effects across a whole population are likely to lead to substantial population‐level benefits.

### Small effects, potential for large impact?

Carey, Ridler, Ford, and Stringaris (2023) point to the negative effect of the COVID‐19 pandemic on adolescent mental health and estimate that even a ‘small’ effect of ~0.1*SD* likely pushed tens of thousands of young people over the threshold at which they begin to need support. Hence, intervention which moved the population in the opposite direction would clearly be worthwhile. Further, small short‐term effects may in some cases accumulate to larger effects over time. Götz, Gosling, and Rentfrow (2022) for example, argue that interventions such as universal free breakfast in schools have small initial effects, which build over time to larger effects on population educational outcomes.

Very long‐term follow‐ups of school‐based interventions are rare, but there is some evidence that intervention in early stages of school can have impacts on outcomes into adulthood, such as continued engagement in education and reduced risks of convictions and incarceration (Reynolds et al., 2007). Effective preventive interventions in childhood and adolescence, whether universal or targeted, are often considered likely to yield greater long‐term economic benefit than those later in life, including through reducing future health and social care burden and economic productivity (Heckman, 2012).

### Can universal school mental health interventions cause harm?

Because school‐based interventions are typically not ‘clinical’ interventions, there is generally less reluctance to introduce them without adequate testing than might be the case for clinical interventions (Foulkes & Stringaris, 2023). In many cases, social interventions are assumed to, at worst, be ineffective but benign. However, they may lead to harms including ‘paradoxical effects’ (i.e. where an intervention worsens the outcome it aims to improve) and ‘externalities’ (i.e. where an intervention causes harm in other outcomes, or for other groups of people) (Bonell, Jamal, Melendez‐Torres, & Cummins, 2015). When considering opportunity cost, a ‘benign’ intervention is harmful, in that it uses resources which might have been directed to something effective. Classic examples of ‘cures that harm’ include ‘Scared Straight’, which aimed to reduce recidivism by exposing young offenders to life in a high‐security prison, but *increased* it (Petrosino, Turpin‐Petrosino, & Finckenauer, 2000). Infant simulator dolls used within schools have been linked to increased teenage pregnancy (Brinkman et al., 2016).

Substantial current debate centres on the potential for universal school‐based mental health intervention to do harm. The MYRIAD study investigated an intervention based on mindfulness in schools, finding no evidence intervention was superior to ‘treatment as usual’ (Kuyken et al., 2022). More concerningly, it was associated with worsening outcomes for young people at high baseline risk (Montero‐Marin et al., 2022). Some recent interventions focused on school‐based cognitive behavioural therapy approaches have also found signals of increases in internalising symptoms (Andrews et al., 2023; Guzman‐Holst, Zaneva, Chessell, Creswell, & Bowes, 2022). Recent evaluation of two universal mental health awareness interventions in England also found increases in emotional difficulties at 9–12 month follow‐up (Deighton et al., 2025).

Foulkes and Andrews (2023) have argued that universal mental health interventions in schools might be causing harm via prevalence inflation. In part, growing mental health difficulties may reflect increased *recognition* and increased help seeking. Increases in estimated prevalence driven by recognition would not represent ‘new’ difficulties. However, a more problematic mechanism of *overinterpretation* is posited. Prevalence difficulties may be increasing through interventions leading to the interpretation of everyday emotions as indicative of disorder. This may lead to responses such as avoidance, creating a self‐fulfilling prophecy. Foulkes and Stringaris (2023) also argue that some school‐based interventions may inadvertently cause rumination.

However, even if some forms of universal mental health intervention cause harm, whether interventions achieve intended or unintended consequences will depend on the specific mechanisms they target. Recent reviews have reached somewhat different conclusions around potential for harm (Guzman‐Holst, Streckfuss Davis, Andrews, & Foulkes, 2025) and benefit (Hayes et al., 2025) in relation to the same narrow subcategory of universal school‐based interventions based on cognitive behavioural therapy. Hence, arguments for resources to be focused on targeted approaches and for the field to move away from universal preventive approaches may be premature. Further, while authors driving this important debate note they are referring specifically to therapeutically informed universal approaches (Andrews & Foulkes, 2025), there are risks of these arguments being over‐generalised to all forms of universal intervention. Nevertheless, these lively current debates make important points regarding a need to be cautious in introducing new interventions into schools and to be mindful that in doing so, we may cause harm.

## Defining school climate as a target for intervention to improve mental health

Much literature has focused on schools as an opportunistic avenue for reaching lots of children for the purpose of delivering new interventions, added on top of what schools already do. For example, a previous practitioner review of mental health interventions in this journal discussed the effects of evidence‐informed behavioural and cognitive behavioural interventions in schools (Paulus, Ohmann, & Popow, 2016). Within Paulus et al.'s review, one contextual factor that is described as important to the successful implementation of such interventions was school climate.

In recent years, however, the field has moved increasingly towards viewing school climate not just as the contextual backdrop for other interventions, but as a target for intervention itself. School climate interventions aim not just to add on new activities, but to change existing everyday practices and processes to create a positive climate. This aligns with wider movements in health promotion towards improving health by improving the places in which people work, live, love, and play (World Health Organization, 1995).

**Figure 1:**
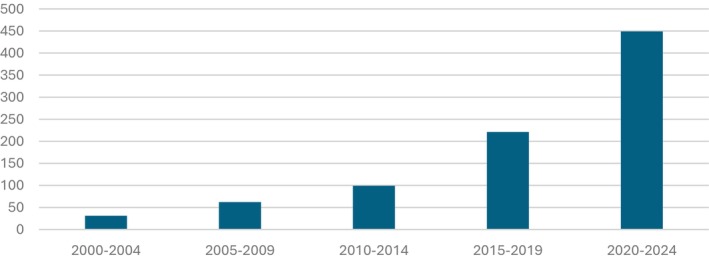
Number of hits in a Medline electronic database search for articles referencing the term School Climate by 5 year period from 2000 to 2024 (‘School Climate’.mp)

A crude indicator of growth in school climate research since the turn of the century is presented in Figure 1. From 2000 to 2004, the term ‘School Climate’ returned 31 hits in Medline, ~ doubling every 5 years, with more than 400 hits in the most recent 5‐year period at the time of writing this article. However, it has remained difficult to define. Some definitions are so inclusive that changing almost anything in a school could be conceived as a change to school climate (Wang & Degol, 2016).

Aldridge and McChesney (2018) conducted a systematic review of the relationship between school climate and mental health and well‐being, defining school climate as including subdimensions of social connectedness, school safety, peer connectedness, and academic environment. In a recent review, 37 measures were identified, which the authors classified into four subdomains: (a) relationships (social relationships, school connectedness, leadership, and culture), (b) environment (school facilities, physical comfort, and cleanliness), safety and discipline (school safety, fairness of rules, bullying and aggression, disciplinary harshness, and general drug use), and academic (academic outcomes, equality of opportunity, engagement, and cohesiveness/competitiveness) (Gonzálvez, Bacon, & Kearney, 2023).

Rudasill, Snyder, Levinson, and Adelson (2018) posit a systems view, applying and extending ecological systems theory (Bronfenbrenner, 1992). This defines school climate in relational terms as ‘affective and cognitive perceptions regarding social interactions, relationships, values, and beliefs held by students, teachers, administrators, and staff within a school’. While many definitions include physical environment and educational instruction (Osher, Neiman, & Williamson, 2020), Rudasill et al. exclude these from their taxonomy. These are viewed as important elements of a school microsystem, separate from school climate.

While many definitions focus on the climate of the school as a whole, some focus on classroom climate (Wang, Degol, Amemiya, Parr, & Guo, 2020). Rudasill et al.'s systems view of school climate conceives classrooms as nanosystems, nested within school systems, and interacting with other features of school climate. Our recent research in Wales finds that variance in child mental health outcomes in primary school is independently associated with measures of school and year group climate (Donaldson et al., 2025).

Hence, consistent definitions remain elusive, with commonalities and differences summarised in Table 1. Perhaps, this is inevitable given the newness of the field. Definitions of complex social phenomena represent constructed frameworks to help shape thinking rather than ‘true’ characterisations of objective realities. Some diversity is to be expected, and perhaps valuable in allowing thinking to evolve, even if this makes synthesis challenging in the meantime. The available definitions, however, share a conceptualisation of school climate as a broad umbrella concept with multiple subdomains. There is some disagreement about whether the physical environment or educational instruction forms a part of the school climate. However, there is consensus across conceptualisations in focus on social relationships among various groups whose actions and interactions make up complex school systems (e.g., students, staff) and on perceived safety within schools. For this review, school climate is considered as the overall atmosphere and feel of a school, shaped by how members of the school community treat and interact with one another, and how safe people feel within it. It intersects with common concepts such as school connectedness and belonging and is sometimes used interchangeably with terms like school environment.

**Table 1:** Examples of subcomponents of school climate from recent reviews and conceptual articles

	Social relationships	Shared beliefs and values	Organisational leadership	Safety	Physical environment	Educational instruction
Wang and Degol (2016)						
Aldridge and McChesney (2018)						
Gonzálvez et al. (2023)						
Rudasill et al. (2018)						

Grey shades indicate the dimensions of school climate included within the articles listed in the left hand column.

Efforts to improve school climate are, by definition, universal, and preventive. This may negate some risks associated with targeting, such as stigma and creation of a ‘missing middle’ who fall just below a threshold. There are some theoretical risks of unintended harm. Structuring school climate so that it entirely removes challenges may arguably impede psychological growth and future well‐being. Navigating challenging encounters and experiences is important for psychological growth (Oshri et al., 2024). However, intervening through changing school climate may avoid some of the potential harms described above. It is hard, for example, to posit a pathway via which improving school climate would lead to prevalence inflation and overinterpretation of everyday emotions.

Efforts to improve school climate will rarely be able to modify every aspect of the broad multifaceted construct at once. Kidger, Araya, Donovan, and Gunnell (2012) early systematic review concluded that progress may be more likely through interventions which target a small number of components of school climate. Before providing an overview of recent systematic reviews, this article will now provide a series of detailed case examples since the turn of the century of interventions which have aimed to improve aspects of school climate, at least in part to improve mental health, over the past 25 years.

## Can interventions improve school climate and mental health outcomes?

This section begins with a detailed overview of several major trials since the turn of the century. These are described chronologically to tell the story of how interventions have evolved over this time and summarised in Table 2. The article will then discuss recent systematic reviews focused on interventions to improve school climate. The overview of trials will focus only on studies that measure both school climate and mental health. The focus is on interventions that aim to achieve system‐wide improvements in climate, rather than, for example, discrete behaviour change interventions for teachers (Okonofua, Goyer, Lindsay, Haugabrook, & Walton, 2022). Systematic reviews have, however, tended to examine effects either on school climate or on child‐level health outcomes, and not both. Hence, the overview of reviews will focus on the effectiveness of interventions to change (aspects of) school climate and the effectiveness of school climate‐focused interventions in changing mental health and other related outcomes (e.g. violence, substance use).

**Table 2:** Summary of school climate intervention trials

Study/intervention	Age of participants at baseline	Follow‐up durations	Effects on school climate	Effects on mental health outcomes^a^	Other (e.g. secondary) effects reported
Bond et al. (2004) Gatehouse Project	Year 8 (~13 years)	~10; 22 and 34 month post‐baseline	No effects on adolescent reported measures of attachment to school	No effect on depressive symptoms	3%–5% risk difference between the two groups in any drinking, any smoking and regular smoking, and friends' alcohol and tobacco use
Sawyer et al. (2010) Beyond Blue	Year 8 (~13 years)	~17 and 29 months post‐baseline	Effects on teacher‐rated, but not adolescent rated, school climate	No effect on depressive symptoms	No significant effects on psychosocial variables
Devries et al. (2015) Good School Toolkit	11–14 years	18 months	Effects on teacher reported school climate reported in follow‐up paper (Kayiwa et al., 2017). No pupil measure	No effect on total SDQ score, but a small effect on school well‐being	The primary aim was to reduce teacher‐to‐child violence, and it was effective in achieving this
Bonell et al. (2018) Learning Together	Year 8 (~12 years)	24 and 36 months post‐baseline	Significant improvements in adolescent rated school climate at 36 months but not 24	Small effect on total SDQ score (0.14*SD*) at 36 months, but not 24 months	Small effects (~0.1*SD*) on bullying (primary outcome), ever smoking, alcohol use, quality of life, contact with police, cyberbullying, aggressive behaviours and truancy
Shinde et al. (2018) SEHER	14 years	8 and 17 months	Significant improvements in adolescent rated school climate at 8 months, sustained to 17 months	Small effect on depressive symptoms at 8 months (0.27*SD*) growing to a larger effect (1.19*SD*) by 17 months	Effects on attitudes toward gender equity, knowledge about reproductive and sexual health, bullying, violence perpetration and victimisation and tobacco use
Cross et al. (2018) Friendly Schools	Grade 8 (~13 years)	Approx. 9 and 21 months	Significant improvements in perceptions of safety in school at first follow‐up, which did not persist to second follow‐up	Small reductions in depressive symptoms (0.15*SD*), anxiety (0.20*SD*) and stress (0.11*SD*) symptoms in first follow‐up, both of which diminished by second follow‐up (to 0.01, 0.02, and − 0.07, respectively).	Small effects also on violence victimisation and perpetration, and loneliness, at first follow‐up. None of which persisted to second follow‐up
Blair et al. (2024) Social and Emotional Education and Development	Two cohorts – 4–5 years and 8–9 years	2, 3, 4 and 6 years post‐baseline	Significant improvements in some measures at 4–6 year follow‐up, but not earlier time points	Significant effect on total SDQ score – 0.27*SD* effect size at primary analysis point of 4 years	Effects on various social and emotional well‐being domains analysed, including self‐esteem, emotional regulation, self‐awareness, social awareness, responsible decision‐making and materialism. Positive impacts on self‐management and decision‐making across multiple time points, others only at a single time points

SDQ, Strengths and Difficulties Questionnaire.

^a^ Effect sizes are noted in brackets where these are reported by study authors.

This does not aim to provide a new systematic review of these highly diffuse literatures but generates a range of insights and questions which future systematic reviews and empirical studies may further explore.

### Case examples of major intervention trials

An early and influential school climate intervention was the Gatehouse Project in Australia (Bond et al., 2004). Drawing on attachment theory (Bowlby, 1979), the intervention involved surveying pupils to understand needs and establishing a school‐based adolescent health team to identify strategies to address these. Schools were supported by an external school liaison team. The intervention did not achieve its primary aims of reducing depressive symptoms. Nevertheless, it improved a range of related behavioural outcomes such as substance use. Rather than overall measures of school climate, the trial examined effects on related constructs focused on attachment to school and conflict with others, finding no evidence of effect.

Also in Australia, the BeyondBlue study had some similarity with the Gatehouse project in intervention and outcomes (Sawyer et al., 2010). Environmental change components included the use of data from staff and students to assess need, and the formation of action groups comprised of staff and research team members to plan actions to improve the school environment. This was accompanied by a curriculum focused on problem‐solving and resilience, building pathways to care and support in the local community and community forums. The intervention was evaluated over a 3‐year period, with a primary outcome of depression, finding no effect. A diverse range of psychosocial outcomes focused on psychological strategies, social support, and relationships in and out of school were measured, as well as school climate from the perspectives of staff and students. Only staff ratings of school climate showed significant improvement, with no change from the perspective of students.

The Good School Toolkit was developed in Uganda as a school‐wide intervention led by teachers, students, and a wider community. The intervention aimed to change school climate through a focus on relationships between students, staff, and the wider school community. A trial in Ugandan primary schools found that it achieved its primary aim of reducing teacher‐to‐child violence and secondary effects on school well‐being, although no effects on pupil mental health, measured by total strengths and difficulties questionnaire (SDQ) scores (Devries et al., 2015). While most evidence discussed in this article focuses on child and adolescent mental health, at least one systematic review identifies school climate as an important predictor of teachers' own mental health (Hooker et al., 2025). Teacher mental health is an important public health issue in its own right and is associated with student mental health (Harding et al., 2019). However, follow‐up analysis of the Good School Toolkit intervention found that improvements in teacher‐rated school climate did not lead to improved teacher mental health (Kayiwa et al., 2017). Work is underway to trial a secondary school adaptation of this intervention (Devries et al., 2024).

Partly inspired by the Gatehouse project, the Learning Together intervention in South‐East England (Bonell et al., 2018) focused on restorative approaches in secondary schools. Restorative approaches focus on building positive relationships and repairing damage to relationships where conflict arises (Lodi, Perrella, Lepri, Scarpa, & Patrizi, 2021). A substantial focus of efforts to improve school climate was via disciplinary policies and practices. Training for staff was accompanied by local data on health and well‐being within the school and externally facilitated action groups. Learning Together placed emphasis on the representation of staff and students in decision‐making, recommending at least six students and six staff included in each group. Groups reviewed data from school surveys and agreed on actions. The intervention also included a social and emotional learning curriculum, though the authors comment that this was weakly delivered and hence likely played a limited role in positive outcomes (Bonell et al., 2019). The primary outcomes were bullying (victimisation and perpetration). These are described by the team as among the most consequential public mental health outcomes, consistent with another annual research review article in this journal (Arseneault, 2018). Reducing bullying perhaps creates feedback loops, with reduced violence likely improving domains of school climate such as perceived safety (López, Lopez‐Nicolas, Lopez‐Lopez, Puente‐Lopez, & Ruiz‐Hernández, 2022; Mori et al., 2021). A range of mental health and well‐being measures were included as secondary outcomes. The team found no effects at 2‐year follow‐up, but significant effects at 3‐year follow‐up. Effects were small (~0.1 of a standard deviation). However, the potential value of the intervention may lie in the number of outcomes it impacted, including bullying, mental health and well‐being, psychosocial functioning, truancy, contact with police, and substance use‐related behaviours (Bonell et al., 2018, 2020). The team has since piloted an adapted version to enhance effects on mental health (Sundaram et al., 2024). A further study is assessing the feasibility of adapting Learning Together for primary schools (Bonell, Legood, Sturgess, et al., 2024).

Published in the same issue of The Lancet as Learning Together, the SEHER intervention in Bihar, India (Shinde et al., 2018) placed substantial emphasis on involving young people in reshaping school climate to improve health. It included school health promotion committees formed of staff, students, and families to review issues raised by students and enact changes, alongside awareness‐raising activities and a suggestion box. This was accompanied by policy changes focused on bullying and substance use, monthly meetings of a group of young people, skills workshops for students, and workshops training teachers in non‐exclusionary discipline. Basic problem‐solving counselling and referrals were provided at the individual level. Two versions were tested against a control group; one with delivery led by lay counsellors, and one in which this was teacher‐led. The lay counsellor‐led version had a large (1.88 standard deviation) effect on young people's perception of school climate, and modest effects on depressive symptoms (0.27*SD*). It achieved improvements in secondary outcomes including bullying, violence, and attitudes to gender equity. By 17‐month follow‐up, effects of the lay counsellor version of the intervention on depression became larger, from 0.27 to 1.19*SD* (Shinde, Weiss, et al., 2020). Change in school climate at 8 months mediated longer‐term effects at 17 months post‐randomisation (Singla, Shinde, Patton, & Patel, 2021).

Notably, the teacher‐led version had no effects. A tendency for limited effect where interventions rely on teacher delivery has been reported for psychological interventions such as group‐based cognitive behavioural therapy (Stallard et al., 2015) and noted in systematic reviews (Werner‐Seidler et al., 2021). This may relate to the need for specialist skills. However, asking teachers to adopt something new, without providing additional capacity and resources, may limit their ability to implement the intervention or lead them to displace other effective activities to create capacity. Process evaluation interviews within the SEHER trial indicated that teachers felt overburdened by academic and non‐academic responsibilities and struggled to integrate this role alongside other responsibilities (Shinde, Khandeparkar, et al., 2020).

The cluster randomised controlled trial of Friendly Schools (Cross et al., 2018) in Australia evaluated a whole‐school intervention focused on the transition from primary to secondary school. Like Learning Together, its primary focus was the prevention of bullying. A school implementation team selected evidence‐based strategies using a staged implementation process according to school needs and capacity. This aimed to create a supportive school culture and positive relationships among staff, children, and families. Transition magazines and classroom curriculum content were provided to children. Newsletters and booklets for parents aimed to enable them to support children through the transition. Small effects were found at the end of the intervention year for depression and anxiety symptoms, loneliness, and bullying as well as perceived safety (the only domain of school climate measured). Hence, the intervention had a beneficial effect through the transition to high school, a period identified as a risk for mental health difficulties (Donaldson, Hawkins, Rice, & Moore, 2024; Donaldson, Moore, & Hawkins, 2023a, 2023b). Effects did not, though, persist to follow‐up 1 year later.

The Social and Emotional Education and Development (SEED) intervention in Scotland, UK (Blair et al., 2024), was also inspired by the Gatehouse Project. Two cohorts, aged 4–5 or 7–8 at baseline, took part. The intervention used school surveys with children on social and emotional well‐being and school culture, benchmarked against averages of all schools, to understand needs. Staff worked with researchers and an educational psychologist to identify and implement solutions. A resource guide summarising interventions and their evidence base was provided to match solutions to issues identified. Children were followed 2, 3, and 4 and 6 years after baseline. At follow‐up 1, cohorts were still in primary school, and the intervention achieved an effect on mental health (i.e. total SDQ score) of between 0.29 and 0.36 standard deviations. The 4‐year follow‐up, at which point the older cohort had transitioned to high school, was pre‐specified as the primary analysis. At this point, differences between intervention and control groups were maintained in analyses including all available data at each time point, although in ‘complete case’ analysis a difference in the expected direction fell short of significance. Effects diminished to a small extent by very long‐term follow‐up, though remained in the anticipated direction.

### Emerging lessons from case study intervention trials

#### Interventions that change school climate (from young people's perspective), can improve mental health

While not all have worked, it is evident from these examples that interventions which improve school climate (from the perspectives of young people) *can* improve mental health (Blair et al., 2024; Bonell et al., 2018; Shinde et al., 2018). Conversely, interventions which did not achieve significant improvements in school climate measures tended not to improve mental health (e.g. Bond et al., 2004; Sawyer et al., 2010). While school climate is commonly conceptualised as an aggregation of the experiences and perceptions of all actors within a school community, few studies have examined impacts from staff and student perspectives. Where staff perspectives are included, change in perceived school climate did not translate as reliably to improved child mental health. For example, Sawyer et al. (2010) found no improvement in adolescent perception of school climate or mental health, despite improvements from teachers' perspectives. Devries et al. (2015) found no effect on child mental health despite improvements in teacher‐rated school climate.

#### Effective interventions have several characteristics in common

Comparing and contrasting interventions which have or have not worked (at least in relation to mental health outcomes) is often difficult for complex multifaceted interventions, such as these, given the tendency for structured intervention description tools not to be consistently used (Hoffmann et al., 2014). Some apparent similarities and differences are likely consequences of how interventions are boiled down to brief descriptions. Methods such as Intervention Components Analysis may be beneficial in rigorously identifying the similarities and differences between interventions (Rizzo et al., 2023), although these also depend on how interventions are reported.

Notwithstanding these challenges, common characteristics across interventions that have shown good effects on mental health are summarised in Table 3.

**Table 3:** Common characteristics of interventions with positive effects on school climate and mental health

Intervention characteristic	Interventions
External support from beyond the school system for implementation of change	Learning Together, SEHER, Friendly Schools, SEED
A focus on reviewing and revising existing policies and practices to make contextually driven changes, rather than just ‘adding on’ new interventions	Learning Together, SEHER, Friendly Schools, SEED
Revising disciplinary policies, and promoting alternative forms of discipline beyond punishment	Learning Together, SEHER
Positive relationship building between students and between staff and students	Learning Together, SEHER, Friendly Schools, SEED
Emphasis on preventing interpersonal violence, including bullying	Learning Together, SEHER, Friendly Schools
Mechanisms for young people to feedback on current assets and problems, and into decision‐making regarding priorities for change and their implementation	Learning Together, SEHER, Friendly Schools, SEED
Classroom curriculum components or skills workshops for young people.	Learning Together,^a^ SEHER, Friendly Schools

^a^ Curriculum component described as weakly implemented and unlikely to be responsible for effects.

By definition, complex interventions are greater than the sum of their parts (Moore et al., 2019), with components acting in configuration (and in interaction with their contexts) to affect change. Any one aspect will rarely be responsible for observed effects, but differences may lie in the configuration of these. For example, a difference between Learning Together and Gatehouse emphasised by the authors of the former is that it includes a curriculum component, which perhaps interacted with environmental change components to amplify effects. However, authors also note that this component was weakly implemented (Bonell et al., 2019).

Further, some of these same characteristics appear to some extent in interventions which did not achieve effects on mental health. Hence, in some cases, effectiveness is perhaps driven by the qualitative nature of components rather than just presence or absence. For example, the Gatehouse Project (Bond et al., 2004) recommended including student representatives within school action groups, likely as one member group among many. However, more recent interventions often placed deeper emphasis on student involvement, with Learning Together, for example, recommending a more equal balance of staff and students within groups.

Progress over the past 25 years has commonly been achieved by building on and refining principles from interventions which did not work (at least in terms of affecting mental health outcomes), rather than building from scratch (Birrell et al., 2025). Trials such as BeyondBlue (Sawyer et al., 2010) attributed lack of effect in part to implementation challenges. Although effective interventions have described weak implementation of some elements, perhaps later studies have taken onboard lessons from earlier trials and improved implementation. Hence, whether the difference in effects relates to differences in implementation fidelity or context, or finer details of how broad activities are operationalised, remains challenging to disentangle.

Importantly, while effects are often small, there is no evidence from these studies of paradoxical effects on mental health. Such harms, which are increasingly described for awareness and skills‐based universal interventions in schools, are perhaps less likely where a major focus is on creating a positive climate.

#### Similar interventions can work across different contexts, although effect sizes vary substantially

As well as differences in whether interventions work, there are important differences in effect sizes. For example, SEHER appeared to have larger effects on mental health than other effective interventions. There is a need for caution in comparing effects in very different contexts (Moore et al., 2021). Perhaps differences between the novel intervention and usual practice were larger in India than in the UK or Australia. For example, while approaches to behaviour policy in England have been described as the most authoritarian of the UK nations (Donnelly & Brown, 2022), Bonell et al. (2019) found that several participating schools used restorative approaches already. In many schools in India, some studies indicate that school discipline tends to be highly authoritarian (Tiwari, 2019). Providing school staff with alternatives to punitive discipline, and support to enact these, may have more potential for effects in some contexts than in others. Further, schools in Bihar, India, typically have student‐to‐teacher ratios more than double those in the UK (Education for all in India, 2023). Hence, additional resources provided by the lay counsellor intervention perhaps represented a large relative increase in capacity to support improvements. Nevertheless, that these similar interventions achieved impacts on school climate and mental health across very different contexts represents a substantial leap forward in our understanding of their potential (Ameratunga, Clark, & Banati, 2018).

Much of the school climate literature derives from higher income countries, with notable exceptions including the Good School Toolkit and SEHER studies described above. There is a need to continue to build understanding of the transferability of evidence on school climate interventions across diverse social, cultural, and economic contexts. Newly developed school climate interventions in countries such as South Africa and Nepal (Laurenzi et al., 2024) have yet to be tested.

#### Sequencing of effects on school climate and mental health varies

Most studies discussed here measured outcomes at more than one time point, although, as indicated in Table 2, choices of time point varied. As well as differences in effect size, these indicate notable differences in how impacts on mental health and school climate played out over time. For Learning Together, neither mental health outcomes nor school climate improved at 2 years, with effects evident only at the 3‐year follow‐up. In SEED, perceptions of school climate showed evidence of improvement only in long‐term follow‐ups, after mental health improvements had become observable (Blair et al., 2024). Hence, this sequencing may be more consistent with improving school climate through improving mental health, rather than the other way around. In SEHER, large improvements in perceptions of school climate were observed at the 8‐month follow‐up (Shinde et al., 2018), mediating change in mental health outcomes at the 17‐month follow‐up (Singla et al., 2021). By contrast, in Friendly Schools, effects at the end of the intervention year were observed but did not persist 1 year later. Hence, all studies provide evidence that interventions can improve school climate and mental health outcomes, as well as a diverse array of risk behaviours. However, arguably, only SEHER provides direct evidence that such interventions improve mental health *by* improving school climate.

#### School climate interventions can influence a broad range of outcomes

Another common feature of effective school climate interventions has been that they achieve effects across a broad range of related outcomes. Indeed, early interventions such as the Gatehouse Project were effective for outcomes such as substance use despite a lack of effect on depression (Bond et al., 2004). Outcomes measured vary across intervention studies, but interventions have achieved a positive influence on outcomes from contact with police and truancy to smoking uptake (Bonell et al., 2018).

Theoretically, this influence upon multiple outcomes makes sense if we conceptualise school climate as an upstream pathway toward multiple outcomes. Jamal et al.'s (2013) review of qualitative studies highlighted a tendency for young people at the margins of their school community to engage in high‐risk or antisocial behaviours as an alternative source of bonding and identity. As Emler and Reicher (1995) argue, antisocial behaviour is often socially motivated by a desire to manage reputation, an important development task of adolescence. Hence, in improving school climate, we may remove the need for young people to engage in behaviours such as violence and substance use, or to find community in unhealthy places. Perspectives from sociology and group psychology have an important role to play in shaping group dynamics in ways that recognise and address reputation and identity needs and create a positive culture which avoids pushing groups of young people to the margins. Effective interventions, such as Learning Together and SEED, have moved toward sociological perspectives focused on how interactions among members of school communities create positive or negative environments (Markham & Aveyard, 2003), while earlier interventions (Bond et al., 2004) drew more on individual or dyadic theories.

Single issue interventions have to date tended to dominate the school health literature, including those which engage with social processes and group dynamics to an extent. For example, an effective intervention for preventing smoking by shifting norms within schools (Campbell et al., 2008) inspires a similar intervention for drug use (White et al., 2017) and another for gambling (Miller et al., 2025). While single issue interventions have a place, no school will have the capacity to deliver a separate intensive intervention for every outcome (Moore et al., 2019). Interventions focused on a single issue may sometimes have a limited shelf life, as norms for specific behaviours shift over time. However, where fundamental social processes, such as social connection and feelings of safety and belonging, can be supported effectively through positive school climate, this is arguably likely to provide durable benefits.

Arguably, interventions which also improve ‘core’ school outcomes such as attendance and educational attainment may be more likely to be taken up. Bonell et al. (2018) found an effect of Learning Together on truancy, and further work indicates an effect on GCSE (UK end of school academic qualifications) performance (Wigelsworth, Thornton, Troncoso, Humphrey, & Black, 2023). To date, few studies move toward understanding whether interventions can have simultaneous effects on outcomes of importance to schools, such as attendance and attainment.

Moving towards approaches which target upstream causes of multiple health outcomes is, of course, a challenge where interventions are evaluated within a traditional medical model paradigm that demands a single primary outcome, which is poorly suited to complex social interventions (Moore et al., 2019; Skivington et al., 2021). Nevertheless, this remains an important direction for research.

### Evidence from recent systematic reviews

Thus far, we have considered several case examples of studies which examine the effects of interventions on school climate and mental health. Systematic reviews have to date tended to examine one or the other of these issues: whether interventions can improve school climate, or whether school climate‐focused interventions can improve health and well‐being outcomes.

Perhaps the most comprehensive suite of early systematic review evidence on school climate (or school environment) interventions to date is by Bonell, Jamal, et al. (2013). Their reviews did not focus solely on mental health but on a broader range of health and well‐being‐related outcomes. The team concluded that there was evidence for the potential of school climate interventions to improve health, but that this was not definitive. A linked meta‐ethnography (Jamal et al., 2013) concluded that behaviours such as violence and substance use were often a strong source of status and bonding for pupils who feel marginalised or unsafe at school. Positive relationships with teachers appeared critical in promoting student well‐being and limiting risk behaviour. However, certain aspects of schools' organisation and policies were associated with an increasing likelihood that students would seek identity and bonding via risky behaviours, with implications for well‐being and mental health. The team also reviewed theories used to explain how the school environment impacts on student health (Bonell, Fletcher, et al., 2013), noting few theoretical perspectives that focus on the role of institutional processes in shaping interactions within schools and a tendency for reliance on individual‐level theorising.

More recent systematic reviews have focused on questions such as the effects of school‐wide interventions on measures of school climate from teacher and student perspectives (Charlton, Moulton, Sabey, & West, 2021); the effectiveness of interventions to build school belonging in adolescents (Allen et al., 2022); the role of school connectedness interventions in preventing depression and anxiety (Raniti, Rakesh, Patton, & Sawyer, 2022), and the impacts of structural and cultural factors and school climate interventions on mental health (Troy et al., 2022); the role of whole‐school interventions in mental health and related risk behaviour outcomes (Lekamge et al., 2025); and the role of a subset of whole‐school interventions which involve a substantial environmental focus on elements of school climate such as students' commitment to one another and the school community (Melendez‐Torres, Ponsford, Falconer, & Bonell, 2023). A summary of these reviews is provided in Table 4.

**Table 4:** Overview of systematic reviews published in the last 5 years on the effects of school‐based interventions on school climate, or related subdimensions, and mental health and related outcomes

References	Primary review question	Number and type of studies	Review conclusions
Charlton et al. (2021)	To what extent do school‐wide interventions influence measures of school climate?	16 experimental studies and 10 quasi‐experimental studies of school‐wide interventions with school climate as an outcome measure	While quality of existing literature is limited, findings support a conclusion that school‐wide interventions can have important effects on school climate. Effect estimates ranged from −0.29 to 1.69 for staff perceptions, and from 0.03 to 1.93 for student perceptions
Allen et al. (2022)	What is the impact of school‐based interventions on school belonging for adolescents aged 12–19?	22 articles evaluating 19 interventions. Included studies which measured school climate with belonging as a subcomponent	Most reviewed studies reported effectiveness of school‐based interventions for enhancing school belonging. Effect size estimates ranged from 0.13 to 0.85 where reported. Successful interventions targeted students' strengths and promoted positive staff and student interactions. Interventions. Effects on mental health were also found in some studies where measured as an outcome
Davies et al. (2025)	What is the effectiveness of interventions to Improve connectedness, belonging, and engagement in secondary schools?	16 randomised controlled trials (11 included in meta‐analysis)	Overall, interventions had a large effect on measured outcomes, with a pooled effect size of 1.06 (Hedge's *g*). Interventions which combined universal, targeted or specialist elements, or which targeted ecological and environmental factors were effective. Those which targeted individual behaviour change or intrapersonal factors were not
Lekamge et al. (2025)	Effects of whole‐school interventions on mental health and risk behaviours for adolescents (i.e. age 12–18)	28 controlled studies	Whole‐school interventions appear to have significant effects on tobacco smoking and on cyberbullying/cyber‐aggression. However, no significant effects on direct measures of mental health symptoms or other forms of violence and substance use were found. A pooled effect size of −0.26 was found for depression indicating small reductions, but the estimate intersected the null
Melendez‐Torres et al. (2023)	Effects of interventions promoting school commitment to school community on violence and substance use	18 randomised controlled trials	Pooled analyses indicate small but significant effects of interventions changing the school environment (pooled ORs 0.79–0.85) to promote student commitment to school community on both violence and substance use
Raniti et al. (2022)	The prospective relationships between school connectedness and depression and anxiety, and the effect of interventions designed to improve school connectedness on depression and anxiety, in young people aged 14–24 years	36 studies were included, of which only two were interventions, and 34 were longitudinal studies of the relationship between school connectedness and depression	Most studies found a significant protective relationship between higher levels of school connectedness and depressive and/or anxiety symptoms. Depression was more commonly measured than anxiety. Both intervention studies improved depression
Troy et al. (2022)	What is the impact of structural and cultural factors and interventions within educational settings on promoting positive mental health and preventing poor mental health?	62 studies, including 25 RCTs, 11 non‐randomised controlled studies, 11 qualitative studies, 8 cohort studies, 6 mixed method studies and 1 case control study	Studies examining positive school culture, teacher training and parent involvement in school mental health activities found predominantly positive results for student mental health

#### Reviews focused on the effects of interventions on school climate

Charlton et al. (2021) reviewed 26 intervention studies focused on the effects of school‐wide interventions on school climate. They concluded that while there continue to be relatively few good quality studies, there is an increasing body of evidence that teachers' and young people's perceptions and experiences of their school's climate are amenable to change through school‐wide interventions. Most studies measured school climate from either the perspective of teachers or young people, with only a small number including both perspectives. Effect sizes for change in teacher perceptions of school climate ranged from small negative effects to a large positive effect (−0.29 to 1.69), while effects on student perceptions ranged from 0.03 to 1.93.

Allen et al. (2022) found that interventions focused on building students' strengths and promoting positive interactions between students, and between school staff and students, were effective in building a sense of belonging. Where studies reported effect sizes (*n* = 13), these ranged from 0.13 to 0.85. While their focus was on belonging, the authors note that many studies included global school climate measures, rather than separating belonging from other aspects of climate.

Most recently, Davies et al. (2025) reviewed 16 trials of interventions to improve belonging, connectedness, and engagement in secondary schools. Overall, interventions showed a large‐pooled effect of 1.06. Interventions that were based on ecological and environmental approaches were effective. However, approaches solely targeting cognitive or emotional intrapersonal factors were not.

#### Effects of school climate interventions on mental health and related outcomes

Raniti et al. (2022) reviewed interventions and a non‐intervention literature focused on school connectedness. School connectedness was defined as students' thoughts, feelings, and behaviours towards their school environment and conceptualised as a component of school climate. The two intervention studies reviewed included SEHER, discussed above, and one focused specifically on preventing depression in transitioning to high school for young people with elevated depression risk (Blossom, Adrian, Vander Stoep, & McCauley, 2020). Both found preventive effects. Raniti et al. reported that non‐intervention studies typically reported longitudinal relationships between school connectedness and later depression and anxiety, with the former measured more often than the latter.

Troy et al. (2022) reviewed a range of intervention and non‐intervention literature, focusing on the impacts of structure and culture of school systems and implications for mental health. The review concluded that, while study quality was often limited and effect sizes poorly reported, interventions based on positive school culture, teacher training, and parent involvement in school mental health activities showed some evidence of promise for student mental health. While, as described, definitions have differed in their inclusion or exclusion of the physical environment, this review identified four evaluations of physical environment interventions, finding that exposure to nature in school settings and providing access to outdoor green space had benefits for mental health. This is perhaps consistent with emerging movements toward the use of ‘forest schools’ in the UK (Garden & Downes, 2023), and recent systematic review evidence on associations between access to nature within a school's physical environment and health and development (Díaz‐Martínez et al., 2023; Meng, Zhang, & Wang, 2023).

Two recent reviews focus specifically on the effects of ‘whole‐school’ interventions in adolescence. Lekamge et al. (2025) synthesised findings from 28 evaluations of whole‐school interventions which align with the WHO Health Promoting Schools framework (i.e., those which include a combined focus on curriculum, creating positive environments, and connections to the wider community). The team found significant effects in meta‐analyses for three outcomes (tobacco smoking, cyberbullying, and cyber‐aggression), with effects on depression or other forms of bullying not significant. The pooled effect size for depression was similar to some of the effects described for school climate interventions above (−0.26), but confidence intervals intersected the null. An overlapping review by Melendez‐Torres et al. (2023) reviewed a narrower subset of whole‐school interventions (but for a wider population of children and young people from 4–18), including only those which focused specifically on improving young people's commitment to their school community. Estimates for individual studies intersected the null, meaning that individual studies provided weak evidence of effect. However, meta‐analyses found that taken as a whole, interventions tended to have small but meaningful effects on violence and substance use outcomes, with pooled odds ratios ranging from 0.79 to 0.85 across outcomes. Effects on mental health symptoms were not a focus of this review, although several studies already discussed which examined these risk behaviour outcomes alongside school climate and mental health symptoms (e.g., Shinde et al., 2018; Bonell et al., 2018) formed part of the review.

#### Summary of key findings from systematic reviews

Alongside the overview of cases of effective and ineffective interventions, reviews provide support for a conclusion that interventions can change various aspects of school climate. There is also tentative evidence that overall, interventions which aim to improve school climate can have small positive impacts on mental health and related behavioural outcomes, such as bullying and substance use. However, systematic reviews tend to group together interventions along differing dimensions of similarity, sometimes leading to slightly variable conclusions. Several authors note a continued shortage of high‐quality evaluation studies. Further, reviews have to date also not fully unpacked the assumption that interventions improve mental health *by* increasing school climate. A need for more work within lower income countries has also been emphasised (Aston, Raniti, & Shinde, 2023). At the time of writing, work is underway to synthesise knowledge about how interventions impact school climate in low and middle‐income countries (Abbott, Shanks, Stanley, & D'Ambruoso, 2024).

## Can school climate intervention reduce mental health inequalities?

There is emerging evidence that school climate can be protective for underserved groups. For example, one recent systematic review of studies on the relationship between school climate and suicidality among LGBT youth found that positive school climate was associated with significantly reduced risk (Ancheta, Bruzzese, & Hughes, 2021). Similarly, a systematic review by Xu and Roegman (2023) found that safe school climate and support from friends and educators were protective factors in the school experience of transgender and gender non‐conforming young people. Ideally, changing school climate would improve outcomes for everyone and reduce inequalities by proving particularly beneficial for marginalised groups.

Perceptions of school climate and health outcomes, tend however to be highly patterned by a range of socio‐economic and other characteristics. Our work from Wales indicates that young people with the lowest well‐being are often those from the poorest family backgrounds attending schools with more affluent intakes. Young people from poorer backgrounds report feeling more accepted and cared for by teachers when attending a school with a lower socio‐economic intake (Moore et al., 2017). This is consistent with ideas from Jamal et al.'s (2013) systematic review, on the role of marginalisation processes in how young people interact with school. Young people whose relative disadvantage is greatest may be at heightened risk of operating at the margins of a community where cultures and norms are shaped around desires and values of a more powerful group. We have found that transitions from one environment to the next (i.e. from primary to secondary) can be associated with worsening outcomes to a greater extent among young people from poorer backgrounds (Donaldson, Morgan, Page, Angel, & Moore, 2024; Moore et al., 2020). The extant literature indicates that disabled young people (Hunt, Radliff, Acton, Bible, & Joseph, 2024), young people from poorer or ethnic minority backgrounds or from minority gender groups (Harwood et al., 2021) tend to experience school climate more negatively than peers. Neurodivergent young people, such as young people with ADHD, are more likely than peers to experience challenges attending schools, with large population‐based studies indicating a sixfold increase in odds of school exclusion (Fleming et al., 2017). Our work with the Child Poverty Action Group found that the more intersections of minoritised identity a person reports, the more negative ratings of school climate tend to be on average (Harwood et al., 2021). There is a substantial history in the sociology of education of considering schools as a microcosm of wider societies, reproducing, amplifying and legitimising societal inequalities (Bernstein, 2003; Bourdieu, 2018). These critiques have only in relatively recent years begun to be applied to understanding school influences on health and health inequalities.

As described, core components of many school climate interventions include responding to local data and involving young people in decision‐making, enabling actions to be tailored to the specific needs of the school community. There is a need for these processes to meaningfully include the voices of underserved groups, or they risk reinforcing a tendency for culture, norms, and values to be shaped around a dominant group, further excluding minoritised groups. School climate interventions may conceivably have the potential to draw young people into a community or drive them to the margins (Fletcher & Bonell, 2013), depending on whether they meet the needs of young people from diverse backgrounds (Forshaw & Woods, 2023).

Evaluations are typically powered around the detection of a main effect, and hence drilling into questions of what worked, for whom, can be challenging within individual studies (Petticrew et al., 2012). However, no intervention works for everyone, and an effect may mask a tendency for large effects for some groups, and small effects (or harms) for others (Pawson & Tilley, 1997). Even though trials tend to be underpowered for meaningful subgroup analyses, it is valuable to report these, so patterns in who interventions work, or do not work, for can be identified through synthesis across studies (Moore, Littlecott, et al., 2015). Troy et al.'s (2022) recent systematic review, discussed above, found that few studies explicitly examined the impact of interventions on mental health inequalities.

Of the case examples discussed above, two trials of effective interventions did examine subgroup effects. Subgroup analyses of the Learning Together intervention (Bonell et al., 2018) found no evidence of differences in effects across socio‐economic subgroups. The intervention, however, had larger effects on mental health for boys than girls. Perhaps, processes of changing school climate favoured changes that met the needs of boys. Similarly, the trial of the SEED intervention (Blair et al., 2024) found no clear differences in effects by socio‐economic background for mental health outcomes. It did find that improvements in liking school, classroom relationships, and perceptions of school climate were limited to ‘non‐deprived’ children, indicating potential for intervention‐generated inequalities. It also mirrored gender differences reported for Learning Together, with larger effects for boys.

We should not over‐interpret exploratory subgroup analyses in a small number of studies and need more data to understand patterns across studies. However, understanding how interventions can improve school climate in ways that benefit all pupils is an important direction for future research in this field. Work is underway, for example, in England to carefully involve autistic young people and young people with ADHD in developing new approaches to creating inclusive school climates (Kakoulidou et al., 2024; Sonuga‐Barke et al., 2024).

## Beyond the randomised trial – What works in the real world?

While recent trials provide reason for optimism that school climate interventions can make a difference to population health outcomes, knowing an intervention ‘works’ in a trial context is one part of the picture. Glasgow et al. (2019) argue that to have real‐world impact, effective interventions need to achieve wide reach and be implemented and maintained. What evidence there is to date indicates that in many cases, this does not happen for school‐based interventions.

SEHER did not continue to be implemented fully in participating schools in the years immediately following the trial (Shinde, Raniti, Sharma, & Sawyer, 2023). Learning Together also experienced challenges with wider scale‐up in a post‐COVID environment where schools struggled to deliver intensive complex programmes, leading the team to seek funding to test simpler school‐based interventions to achieve the same goals (Bonell, Legood, Ponsford, et al., 2024). In a recent systematic review of mental health interventions in schools, March, Stapley, Hayes, Town, and Deighton (2022) identified a range of barriers and facilitators to sustainability, including leadership, staff engagement, characteristics of the intervention, and external systems of support. No identified studies focused on school climate interventions, with most being targeted treatment interventions in schools. Nevertheless, the review found that where interventions led to perceived improvements in classroom climate, this acted as a facilitator of continued delivery. Another review of the sustainment of school‐based health interventions (not limited to mental health) identified 24 studies of 18 health interventions; none were fully sustained following evaluation, although in most cases, some elements were retained (Herlitz, MacIntyre, Osborn, & Bonell, 2020).

In a systematic review of process evaluations on the implementation of school violence interventions, Ponsford, Falconer, Melendez‐Torres, and Bonell (2022) conclude that school leaders are most likely to adopt interventions related to a problem they already know and care about. It is perhaps likely that schools that take part in trials are more persuaded of the need for a given intervention than the general population of schools to whom scale‐up will then be attempted. The small nature of some of the effects observed in most school climate interventions means they are perhaps vulnerable to becoming diluted to zero where interventions are not sufficiently well sustained or scaled or encounter new contextual barriers as they aim to reach a more representative set of schools.

Challenges sustaining school climate interventions over time perhaps include the fact that many require external support for effective delivery. Our research from Wales finds that adopting whole‐school approaches is made challenging by the fact that school staff workloads are so high that adding more to this without taking anything away is challenging (Brown et al., 2025). Werner‐Seidler et al. (2021) in updating their own systematic review of school‐based interventions for anxiety and depression, noted a growing trend towards the use of digital and technological innovations to enable delivery. Whether these can contribute to sustainability is an important direction for research.

However, there are conceptual issues to be understood regarding what it means for a school climate intervention to be sustained over time. For a psychological or curriculum‐based intervention, sustainment might mean that the same intervention is delivered to each new cohort of students coming through the school. However, where the aim is to change the school climate, once new ways of working become normalised, it might be that schools no longer need to keep delivering all aspects of the intervention. Perhaps, rather than asking whether intervention packages continue to be delivered in full, we need to better understand whether and how changes to school climate activated by such interventions are routinised and sustained in the long term, sufficiently to benefit new students coming through the school.

The limited sustainment of evidence‐based interventions in schools to date introduces practical and philosophical questions regarding what evaluation of complex interventions (Skivington et al., 2021) in social institutions such as schools is for. Some argue that the assumption that such interventions can ever be fully reproduced wholesale is unrealistic, and that evaluation is not about testing a ‘programme’ but about testing a ‘programme theory’ (Pawson, 2021). Evaluations may be about building understandings of the mechanisms that work for bringing about change, and in what contexts (Bonell, Fletcher, Morton, Lorenc, & Moore, 2012; Moore, Audrey, et al., 2015; Pawson & Tilley, 1997). Even if the above interventions are never fully reproducible at scale, these give us a useful set of principles, guided by the sum of the evidence base rather than one individual study, which schools can integrate into daily processes and practices. Some of this will require support from actors beyond the school system itself, including support from regional and national governments (Kendziora & Osher, 2016; Margaretha, Azzopardi, Fisher, & Sawyer, 2023).

For example, a key element of some of the successful interventions described above is the assessment of needs based on local data and feedback from young people within the school. In Wales, the government has invested in the creation of data infrastructures to provide schools with local data on needs and assets, with a School Health Research Network involving all secondary schools (Murphy et al., 2021) and a growing number of primary schools (Moore et al., 2024). This aims to provide benefits for all partners, providing the government with national data for surveillance, academic researchers with data for policy and other research (Page et al., 2024), and schools with local data to inform action plans in their setting. Ongoing efforts are extending this model to England (Widnall et al., 2023). This is only one part of these successful interventions. To drive meaningful change in school environments typically requires support from actors outside of the school setting. In Wales (Rothwell et al., 2010), as well as elsewhere in Europe (Buijs, 2009), this is provided via a national network of local healthy school schemes, which employs skilled coordinators who can work with schools to interpret data and develop plans to change their environments. In an increasing number of contexts, such structures are accompanied by government guidance for implementing a ‘whole‐school approach’ to mental health and well‐being (Brown, Van Godwin, Edwards, Burdon, & Moore, 2023). Whether these evidence‐informed elements cohere in a manner that replicates benefits observed in controlled evaluations is an important question for research.

Hence, we are reaching a stage where we can say with some confidence that interventions can modify school climate, and where they achieve this, can improve mental health outcomes for young people. Understanding whether enough of a lasting residue is left after the trial wraps up for real‐world impacts to be maintained, or whether whole education systems can distil enough of the lessons from these interventions into everyday practice to make a difference, is an important next direction for research. This will likely require integration of structures to routinely measure various facets of school climate within and across education systems (Berg, Diffenderffer, & Osher, 2022).

## Conclusions

Schools can positively influence young people's mental health through interventions that improve school climate. While school climate is a complex, multidimensional construct, interventions that target aspects such as staff–student relationships and disciplinary practices have shown promise in improving school climate and mental health outcomes. Although effects are modest, the potential for population‐level impact is significant. School climate interventions may yield benefits across a range of outcomes beyond mental health, including reductions in bullying, substance use, and truancy, and improvements in school engagement and academic performance. This positions school climate as an important upstream target for addressing multiple public health and educational goals simultaneously. While at the time of writing there are lively debates around the potential harms of universal school‐based interventions, there is little evidence that school climate interventions cause harm. Such debates are important but should be carefully framed to avoid over‐generalisation and dissuading schools from adopting approaches which have substantial potential benefits.

Nevertheless, challenges remain. There is limited evidence on how school climate interventions affect mental health inequalities. Where subgroup analyses have been conducted, some findings suggest that interventions may benefit certain groups more than others, raising tentative concerns about intervention‐generated inequalities. Future research must prioritise equity‐focused intervention development, ensuring the voices of underserved groups are reflected in actions to change school climate and evaluations. Second, sustainability and scalability remain uncertain.

To ensure long‐term and equitable impact, policy, and practice could shift towards embedding principles of effective interventions into everyday school processes. This could include national and regional infrastructures that support schools in assessing and improving their climate, sustained funding for external facilitation and workforce development to support implementation and embedding school climate improvement into statutory guidance and accountability frameworks. Schools will need support from wider systems to do this but should prioritise student involvement in designing and implementing school climate initiatives, focusing on revising existing policies and practices (e.g., disciplinary systems) rather than layering on new programmes, and using local data to tailor actions to the specific needs of the school community. Those tasked with further building this evidence base need to conduct long‐term follow‐ups to assess the durability of intervention delivery and effects, better understand how improvements in school climate translate into mental health outcomes, evaluate how interventions can be adapted and sustained within diverse educational systems and cultural contexts, and understand models for integrating school climate improvement into whole‐system reform efforts.

## Ethical considerations

As this is a review article, no ethical review was required.

**Key points**

**What's known**

- Schools are important settings for interventions to improve mental health. However, many interventions are ineffective, or might even be harmful.
- Increasing attention is being paid to school climate, and interventions to improve school climate, in improving mental health.

**What's new**

- School climate is difficult to define, but definitions overlap in their focus on social relationships among the members of a school community and safety in school.
- Several large recent trials have found that interventions in schools can improve school climate, and improve population mental health, as well as an array of risk behaviours and other outcomes.

**What is relevant**

- Less is known about how school climate interventions can reduce inequalities in mental health.
- There are also ongoing questions regarding the scalability and sustainability of school climate interventions, and hence, translation into meaningful population‐level impact.

## Data Availability

Data sharing not applicable to this article as no datasets were generated or analysed during the current study.
